# Human Serum Albumin-Based Nanoparticle-Mediated *In Vitro* Gene Delivery

**DOI:** 10.1371/journal.pone.0107603

**Published:** 2014-09-17

**Authors:** Monica Langiu, Miriam Dadparvar, Jörg Kreuter, Mika O. Ruonala

**Affiliations:** 1 Center for Membrane Proteomics, Goethe University, Frankfurt am Main, Germany; 2 Institute of Pharmaceutical Technology, Goethe University, Frankfurt am Main, Germany; Martin-Luther-Universität Halle-Wittenberg, Germany

## Abstract

The genetic treatment of neurodegenerative diseases still remains a challenging task since many approaches fail to deliver the therapeutic material in relevant concentrations into the brain. As viral vectors comprise the risk of immune and inflammatory responses, human serum albumin (HSA) nanoparticles were found to represent a safer and more convenient alternative. Their ability to cross the blood-brain barrier (BBB) and deliver drugs into the brain in order to enhance gene-based therapy has been previously demonstrated. The present study deals with the development of pGL3-PEI-coated HSA nanoparticles and subsequent *in vitro* testing in cerebellar granular and HeLa cells. The luciferase control vector pGL3 was chosen as reporter plasmid encoding for the firefly luciferase protein, linear polyethylenimine (22 kDa) as endosomolytic agent for enhancing the cells’ transfection. Studies on particle characteristics, their cellular uptake into aforementioned cell lines and on subcellular localisation, and transfection efficiency in the cerebellar cells proved the feasibility of nanoparticle-based gene delivery.

## Introduction

In numerous neurodegenerative diseases the onset and course are attributed to a gene defect that results in the production of a functionally modified protein [1]. Complete removal or reconstitution of the affected protein could result in the attenuation or remission of disease symptoms by either restoring the original function, or by inhibition of the latter [2]. Present molecular biology technologies provide ample of possibilities to achieve this *in vitro* through transient transfection of cells with either plasmid cDNA encoding for the functional protein, or small interference RNA, as the most commonly used forms. Due to the highly selective endothelial cell layer of the brain blood capillaries, the blood-brain barrier (BBB) which maintains the brain homeostasis and protects the central nervous system from assaults, these materials will not be transported *per se* into the brain, and thus will not be able to transfect neuronal cells in the intact brain [3], [4], [5]. In earlier reported studies, genetic material has been delivered beyond the BBB by microinjection of adeno-associated viruses (AVV) directly into the brain tissue [6], [7], [8], [9]. Studies performed on rodents and non-human primates suggest that the gene therapy approach itself might be successful, but also demonstrate the multiple operational risks associated with these procedures [10], [11]. Unfortunately, the more convenient and safer intravenous treatment of brain associated disorders remains a challenging task as the application of injected genetic treatments is usually thwarted by their short survival in the bloodstream due to the presence of serum nucleases [12].

Recent developments in pharmaceutical nanotechnology have shown the suitability of using biodegradable nanoparticles as transport vehicles for antibodies, growth factors, functional recombinant enzymes, as well as genetic materials across cellular plasma membranes and the BBB [13], [14], [15]. Nanoparticles made of human serum albumin (HSA) provide the benefits of targeting the neuronal cells beyond the BBB without any toxicity or immune response [15], [16]. The preparation of HSA nanoparticles using desolvation in ethanol described earlier by Langer et al. [17], produces globular aggregates with a size of a few hundred nanometers in diameter. The biologically active molecules can either be entrapped inside the nanoparticle core during their preparation, or bound on the surface of previously manufactured particles. Further covalent surface modifications, such as the attachment of apolipoproteins, enhance the brain targeting after intravenous (i.v.) injection without the opening of the tight junctions of the BBB [14], [15]. This feature provides additional flexibility in order to target certain cell types within the brain. So far the HSA nanoparticles have successfully been used to deliver a variety of hydrophilic drugs over the BBB, such as loperamide, dalargin, and doxorubicin [18], [19]. Although nanoparticles appear to represent a promising tool for drug delivery to the central nervous system, very little is known about their suitability to carry genetic material. To date, similar nanoparticle systems have successfully been described to enable the transfection of human epithelial kidney 293 cells as well as MCF-7 breast cancer cells [12], [20]. In the present study the eligibility of plasmid cDNA-polyethylenimine (PEI) coated HSA nanoparticles to mediate transfection and protein expression in cells of neuronal origin was tested. The cationic PEI-polymer, a strong DNA complexing agent featuring a high endosomolytic activity was used to enhance the intracellular processing of the genetic material [21]. The newly developed nanoparticle system was characterised using photon correlation spectroscopy and morphological analysis; its *in vitro* uptake and degradation were analysed using FACS, biochemistry, and image analysis. Biochemical measures were taken into account to determine the cDNA-PEI-coated nanoparticles’ *in vitro* biodegradability. Finally, the *in vitro* transfecting efficiency of the cDNA-PEI-coated nanoparticles proved the feasibility of nanoparticle-mediated gene delivery system within a neuronal stem cell model and sets the path for *in vivo* studies.

## Methods

### Amplification of pGL3 plasmid

The pGL3 control vector plasmid (Promega GmbH, Mannheim, Germany), encoding for the firefly luciferase, was transformed into chemical competent *E.coli* cells (One Shot Top 10, Life Technologies, Darmstadt, Germany) according to the manufacturer’s protocol. Bacterial colonies were amplified in ampicillin-containing medium, and the plasmid cDNA was purified using the QIAGEN Plasmid Plus Midi Kit (QIAGEN GmbH, Hilden, Germany) according to the manufacturer’s high yield protocol. The plasmid content was determined with a Nanodrop 2000™ spectrophotometer (Thermo Scientific, Wilmington, USA), and the plasmid cDNA stocks were stored at −20°C.

### Preparation and characterisation of pGL3-PEI-coated HSA nanoparticles

HSA nanoparticles were prepared by ethanolic desolvation as described previously [17]. In short, the protein particles were stabilised by the addition of glutaraldehyde solution in a 2-fold volume of the theoretical amount necessary for crosslinking the free amino groups on the albumin surface [17], [22]. The suspension was kept under constant stirring for 18 h at room temperature. To remove unbound albumin, glutaraldehyde residues, and ethanol, the HSA nanoparticles were purified by three repeated cycles of 10 min centrifugation (16100×g) and redispersion to the original volume of purified water (Centrifuge 5804 R, Eppendorf, Hamburg, Germany). The particles were then diluted 1∶100 in purified water and characterised according to their size, polydispersity index, and zeta potential, by photon correlation spectroscopy (PCS) (Malvern zetasizer nano) with dipcell. The final nanoparticle yield was determined gravimetrically.

pGL3-PEI complexes were prepared according to the manufacturer’s protocol (PeqLab Biotechnologie GmbH, Erlangen, Germany) immediately prior to use. Briefly, equal volumes of plasmid and PEI in 150 mM sodium chloride solutions were mixed 1∶1 and incubated for 15 min (21°C, 650 rpm). The N/P ratio of 6 was calculated according to (1), where N represents the number of nitrogen atoms in the PEI molecule, and P the number of phosphate groups in an average DNA molecule. PEI was provided as a 7.5 mM solution.
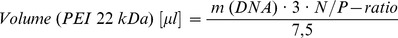
(1)


An aliquot of the complexes was characterised by PCS whereas the rest was conjugated on the surface of HSA nanoparticles (15 min, 21°C, 650 rpm). The ratio between pGL3-PEI complexes and HSA nanoparticles was set to 2 µg plasmid DNA per mg nanoparticles. pGL3-PEI-coated nanoparticles used for transfection assays were diluted with cell culture medium prior to use.

### Morphological analysis by scanning electron microscopy (SEM)

Unmodified and pGL3-PEI-coated HSA nanoparticles, as well as pGL3-PEI complexes were diluted with purified water (1∶10). An aliquot was placed on an aluminium sample plate and air-dried at room temperature over-night. In order to obtain electrical conductibility for electron microscopy analysis, the sample plates were sputtered with gold for 45 s under argon gas atmosphere (Automatic sputter coater, Agar Scientific, Essex, United Kingdom). Samples were analysed with a field emission electron microscope with upper detector (EM Hitachi S-45000, Hitachi High-Technologies Europe GmbH, Krefeld, Germany) at 15–25 kV.

### Culture of HeLa and mouse cerebellar granule (Cb) cell lines

The cerebellar granule (Cb) cell line derived from CD1 mice was a kind gift from Dr. Susan Cotman (MGH, Harvard Medical School, Boston, MA, USA) and maintained as described previously [23]. In short, the cells were grown in Dulbecco’s Modified Eagle Medium (DMEM) supplemented with d-glucose, L-glutamine and pyruvate, with the addition of foetal calf serum, penicillin, streptomycin, Geneticin (Life Technologies, Darmstadt, Germany), and potassium chloride (Sigma Aldrich, Munich, Germany). The cells were maintained in a humidified incubator set to 33°C under 5% CO_2_ atmosphere. The HeLa cell line (LGC Standards GmbH, Wesel, Germany) was grown in DMEM supplemented with glucose, foetal calf serum, penicillin and streptomycin in a humidified cell incubator set to 37°C under 5% CO_2_.

### Fluorescence-activated cell-sorting (FACS)

The effect of nanoparticle type, concentrations, incubation temperature and time on the nanoparticle uptake was investigated using FACS analysis. For this, Cb cells were seeded into 24 well plates at a density of 10^5^ cells/well and incubated at 33°C for 24 h prior to the experiment. Unmodified HSA nanoparticles at a concentration range from 0.1 to 1.0 mg/ml were suspended into cell culture medium, added on the cells, and incubated at 33°C for 18, 24, 48, or 72 h. To test the effect of temperature, equally treated Cb cells were incubated either at 4°C or at 39°C [13], [23]. The effect of nanoparticle formulation was investigated by comparing the uptake efficiencies of 0.25 mg/ml of unmodified HSA nanoparticles, against equal concentrations of pGL3-PEI-coated HSA nanoparticles. For FACS analysis, cells were washed twice with phosphate buffer (PBS), trypsinised (Life Technologies, Darmstadt, Germany) and resuspended in ice-cold PBS. The characteristic autofluorescence of nanoparticles was quantified using 10^4^ cells per condition in the FL1 channel (FACSCalibur™, BD Biosciences, Heidelberg, Germany).

### Immunofluorescence

Cb cells were seeded on 12 mm round coverslips in 24 well plates (10^4^ cells/well; Greiner Bio-One GmbH, Frickenhausen, Germany), and cultured for 24 h at 33°C. The cells were then incubated with pGL3-PEI-coated HSA nanoparticles at concentrations ranging from 0.1 mg/ml to 1.0 mg/ml suspended in DMEM or with DMEM without nanoparticles for 72 h. Subsequently, cells were washed three times with PBS and incubated for further 24 h with culture medium. The cells were again washed with PBS and fixed with 4% paraformaldehyde in PBS, permeabilized with methanol at −20°C for 5 min, followed by a quenching reaction of the remaining non-reacted aldehyde groups with a 10 minute incubation of 50 mM glycine in PBS. The cells were further incubated for 30 min with 5% bovine serum albumin (Sigma Aldrich, Munich, Germany) in PBS. The pGL3-PEI-coated HSA nanoparticles taken up by the cells were immunolabelled with polyclonal goat anti-HSA antibody (1∶250; Sigma Aldrich, Munich, Germany) and Alexa647 conjugated donkey anti-goat secondary antibody (1∶500; Life Technologies, Darmstadt, Germany). The samples were imaged with a TCS SP5 confocal microscope (Leica Microsystem, Heidelberg, Germany) using a 40x oil objective, and images processed with ImageJ [24].

### Dot blot analysis

Cb cells were seeded in 24 well plates (25×10^3^ cells/well; Greiner Bio-One GmbH, Frickenhausen, Germany), incubated at 33°C for 24 h, and then at 39°C as above, to stop cell division [23]. Cells were then incubated for 24 h with pGL3-PEI-coated HSA nanoparticles (0.25 mg/ml, 24 h) suspended in cell culture medium. Subsequently, the cells were washed with PBS, and incubation continued in fresh DMEM for other 1 to 4 days. Cell samples were dissociated with trypsin, and lysed by using CellLytic M solution (Life Technologies, Darmstadt, Germany) supplemented with a proteinase inhibitor cocktail (Sigma Aldrich, Munich, Germany). The cell lysates were shaken on ice (15 min), and stored at −20°C until usage.

To monitor the degradation beyond cell culture, 0.5 mg HSA nanoparticles were digested with 0.6 U of proteinase K in PBS (pH 8.0) at 37°C [25]. Samples were taken after 10, 20, 45, 60, 75, 120, 150 minutes, or after over-night digestion placed in tubes containing proteinase inhibitor cocktail, and frozen at −20°C. Non-digested HSA-nanoparticles were used as positive control. A 2 µl aliquot of cell lysate or chemically degraded HSA nanoparticles was applied on a nitrocellulose membrane (Thermo Fisher Scientific Inc., Bonn, Germany) as a drop, and air dried for 10 minutes. Membranes were then blocked respectively with 5% bovine serum albumin and 5% milk in Tris buffered saline (TBS) containing polysorbate 20 (Sigma Aldrich, Munich, Germany) at pH 7.5. Non-degraded HSA was detected by incubation with goat anti-HSA antibody (1∶2000) and donkey anti-goat HRP-conjugated antibody (1∶30000, DIANOVA GmbH, Hamburg, Germany). Cell lysate values were normalised against cellular GAPDH amount as determined by HRP-conjugated anti-GAPDH antibody (1∶1000, AbCam plc, Cambridge, United Kingdom). The ECL reactions were performed with home-made ECL reagents and visualised with Chemo Cam Imager (INTAS Science Imaging Instruments GmbH, Göttingen, Germany). Resulting images were processed and quantified by ImageJ [24].

### Luciferase assay

Cb or HeLa cells were seeded into white 96-well plates with flat and transparent bottom (Lonza, Basel, Switzerland) at a density of 5×10^3^ cells/well and incubated for 24 h at 33°C or 37°C, respectively. The Cb cells were then moved to 39°C for another 24 h in order to prevent their cell division [23]. On the next day, pGL3-PEI-coated HSA nanoparticles were added on the cells to a final concentration of 1 mg/ml, and incubated for 72 h. Subsequently, the cells were washed three times with PBS, and the incubation was continued in normal cell culture medium for further 48, 72, or 96 h. The luciferase activity was measured using the Bright-Glo™ Luciferase Assay System (Promega GmbH, Mannheim, Germany), according to the manufacturer’s instructions during a 10 second-interval with a Tecan Infinite 200 microplate reader (Tecan Deutschland GmbH, Mainz, Germany). Cells were also incubated with non-nanoparticle bound pGL3-PEI complex as a positive control. All measurements were performed in triplicate. Well values higher than negative control average + 3x standard deviation, were considered positive.

### Statistical analysis

Statistical analysis was performed using Origin (OriginLab, Northampton, USA). Data was expressed as a ratio against the control group, and the differences between the groups were evaluated with ANOVA-one-way with Tukey post-hoc test. *p* values<0.05 were considered to be statistically significant.

## Results and Discussion

The aim of this study was to develop and test a nontoxic gene delivery system based on human serum albumin nanoparticles. In order to protect the DNA taken up by the cells from degradation by cellular nucleases, enhance endosomal escape, promote the migration to the nucleus, and enhance expression, linear polyethylenimine (22 kDa) was used as a transfection reagent [26]. Pre-manufactured HSA nanoparticles were coated with cDNA-PEI complexes and characterised by PCS. Uptake, degradation and transfection efficiencies of HSA nanoparticles were tested in cerebellar granule cells derived from CD1 mice [23].

### Characterisation of pGL3-PEI coated HSA nanoparticles

**Figure 1:**
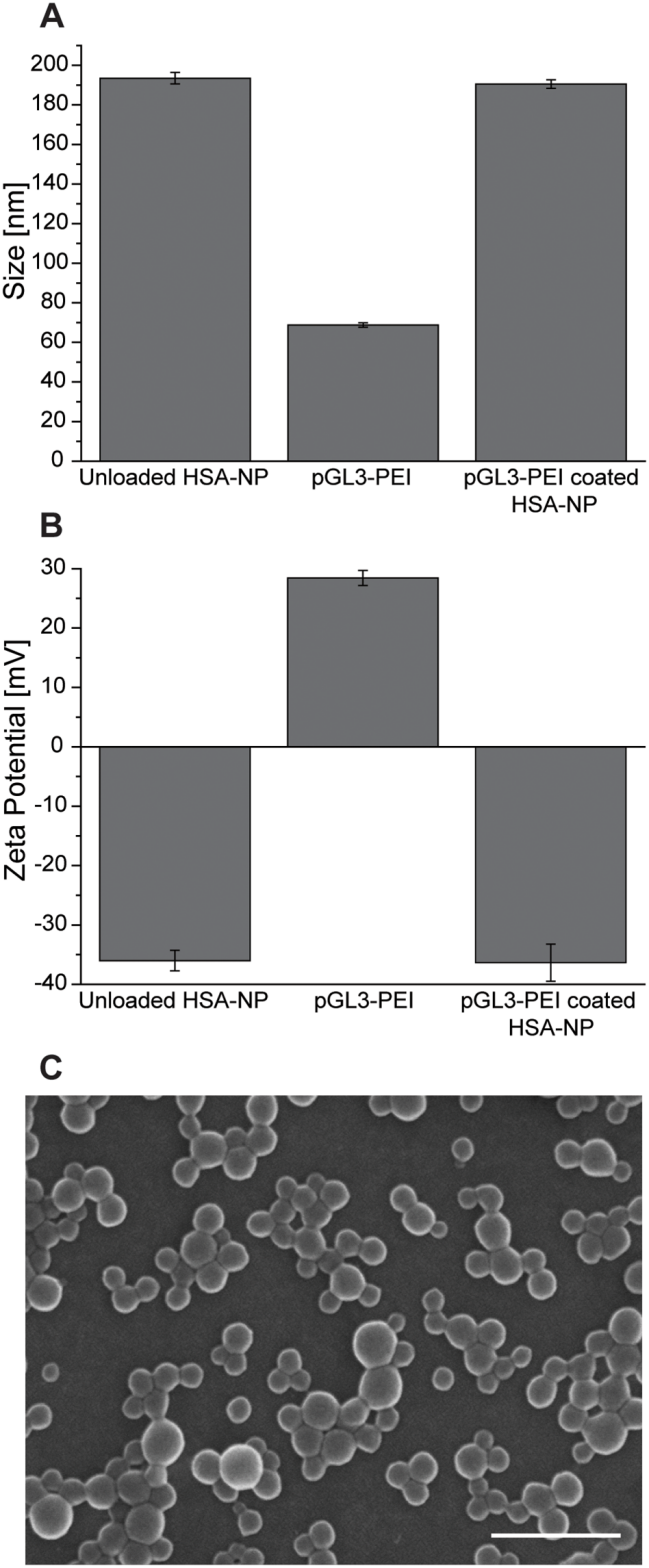
Particle characteristics of pGL3-PEI-coated nanoparticles, unmodified HSA nanoparticles, and pGL3-PEI complexes. Particle sizes [nm] (**A**) and surface charge [mV] (**B**) of unmodified HSA nanoparticles, pGL3-PEI complexes, and pGL3-PEI-coated HSA nanoparticles. Results are shown as mean ± S.E.M. (n = 3). (**C**) Scanning electron microscopy picture of pGL3-PEI coated HSA nanoparticles. Scale bar 700 nm.

HSA nanoparticles used in this study had a size of 191±4 nm, were of a monodisperse size distribution as shown by their low polydispersity index (<0.1), and possessed a negative net charge (−36±5 mV). pGL3-PEI complexes showed a particle size of 67±4 nm with a positive surface charge (28±2 mV). Absorption of pGL3-PEI complexes to unmodified HSA nanoparticles retained the nanoparticles sizes as well as their negative surface charge (Fig. 1A and B), which is vital to prevent agglomeration together with serum proteins in tests in serum-containing media and *in vivo* [27]. Both, unmodified and pGL3-PEI-coated HSA nanoparticles were of spherical form with uniform size distribution as shown by SEM (Fig. 1C). Investigations with agarose gel electrophoresis, and UV-spectrometry on the supernatants of pGL3-PEI complexes after high speed centrifugation and heparin decomplexation as applied earlier [28], [29], or transformation of chemically competent bacteria with the same supernatants revealed the pGL3-PEI complex to be quantitatively bound to the HSA nanoparticles with no free complex or plasmid DNA in the preparations (data not shown).

### Cellular uptake of HSA and pGL3-PEI-coated HSA nanoparticles

**Figure 2:**
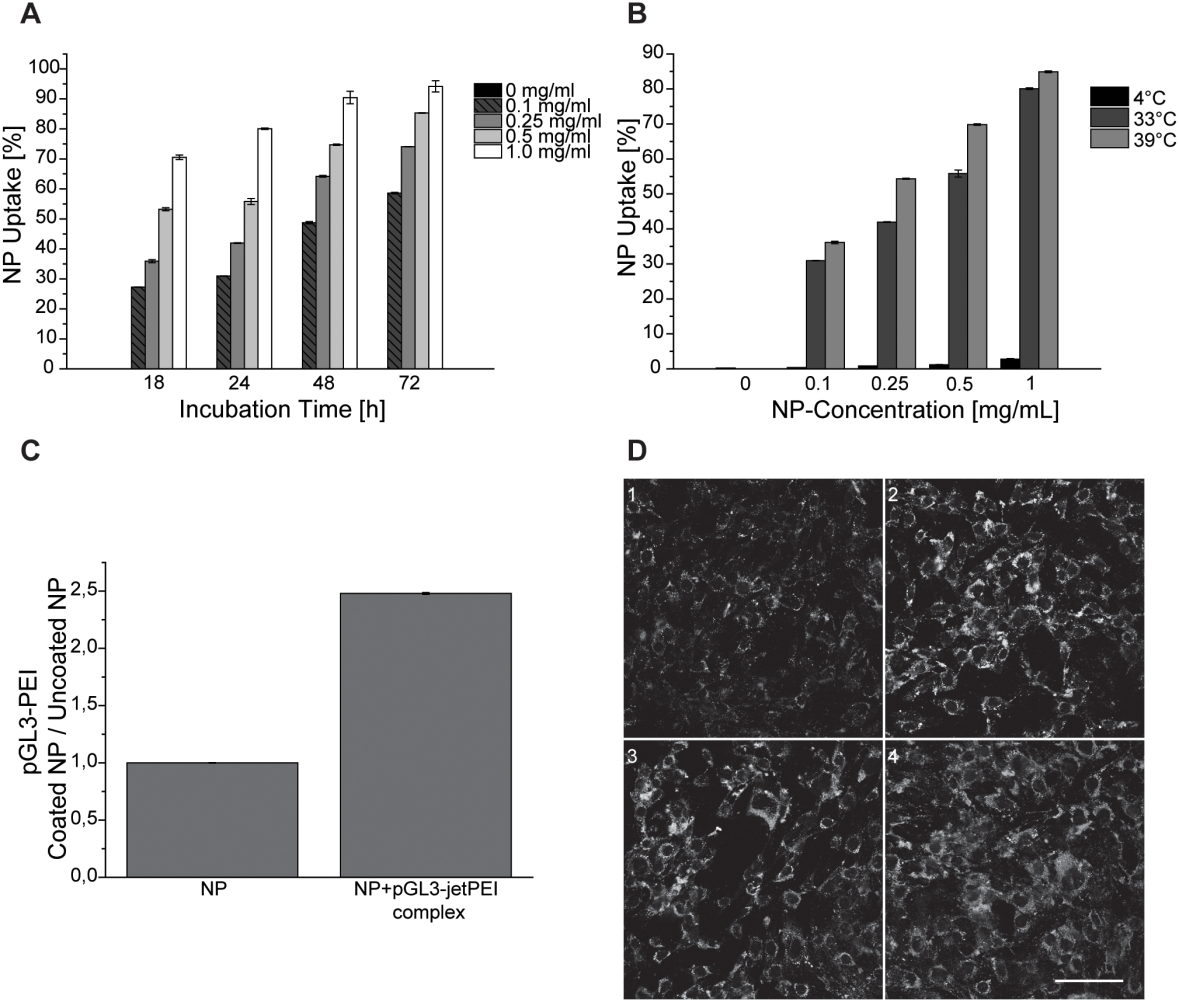
Uptake of HSA nanoparticles and pGL3-PEI-coated HSA nanoparticles in cerebellar granule cells. **A:** Flow cytometry was used to determine the autofluorescence of the HSA nanoparticles in Cb cells. Nanoparticle uptake into the cells increased with higher concentrations of used nanoparticles (0.1 mg/ml to 1.0 mg/ml) and with incubation time (18 h to 72 h). **B:** Temperature dependency of nanoparticles uptake at 4°C, 33°C and 39°C. **C:** Comparison between unmodified and pGL3-PEI-coated HSA nanoparticle uptake in Cb cells. Results are shown as mean ± S.E.M. (n = 3). **D:** Confocal microscopy images represent the uptake of nanoparticles in Cb cells at different concentrations. 0.1 mg/ml (1), 0.25 mg/ml (2), 0.5 mg/ml (3), 1 mg/ml (4). Scale bar 100 µm.

In order to study cell adhesion and cellular uptake of HSA nanoparticles at various conditions, flow cytometry and immunofluorescence microscopy was used. As shown by FACS analysis (Fig. 2A), increasing nanoparticle concentration in cell culture medium as well as prolongation of the incubation time, led to an increase in cellular uptake. For 1 mg/ml HSA nanoparticles and 72 h incubation time, 94%±3% of measured cells showed a shift in the FL1-channel indicating the association of nanoparticles to the cells. The significantly reduced autofluorescence in cells incubated with nanoparticles at 4°C demonstrates the uptake of nanoparticles to occur via an active endocytosis (Fig. 2B) [13]. Additionally, cells were treated with nanoparticles at 39°C to prevent cell division [23]. At this temperature, the average cellular autofluorescence signal increased when compared to that obtained with cells incubated at 33°C. In the subsequent experiments the uptake efficiency of both, unmodified and pGL3-PEI-coated HSA nanoparticles, was compared (Fig. 2C). After 24 h of incubation, the cellular uptake of pGL3-PEI-coated HSA nanoparticles increased over 2.5-fold in comparison to unmodified nanoparticles. This finding can be attributed to the altered surface characteristics known to enforce cell adhesion and cellular uptake [30]. Due to the negative charge of pGL3-PEI-coated HSA nanoparticles, it seems unlikely that the enhanced uptake is mediated by absorptive-mediate endocytosis which has been described as the entry mechanism for cationic albumin nanoparticles [31], although this possibility cannot be completely excluded. In order to verify that the signal arose because of the uptake and not because of surface binding of nanoparticles, Cb cells treated with pGL3-PEI coated HSA nanoparticles were visualised using immunofluorescence (Fig. 2D). The micro images show a clear subcellular localisation for the nanoparticles.

### *In vitro* degradation of pGL3-PEI-coated HSA nanoparticles

**Figure 3:**
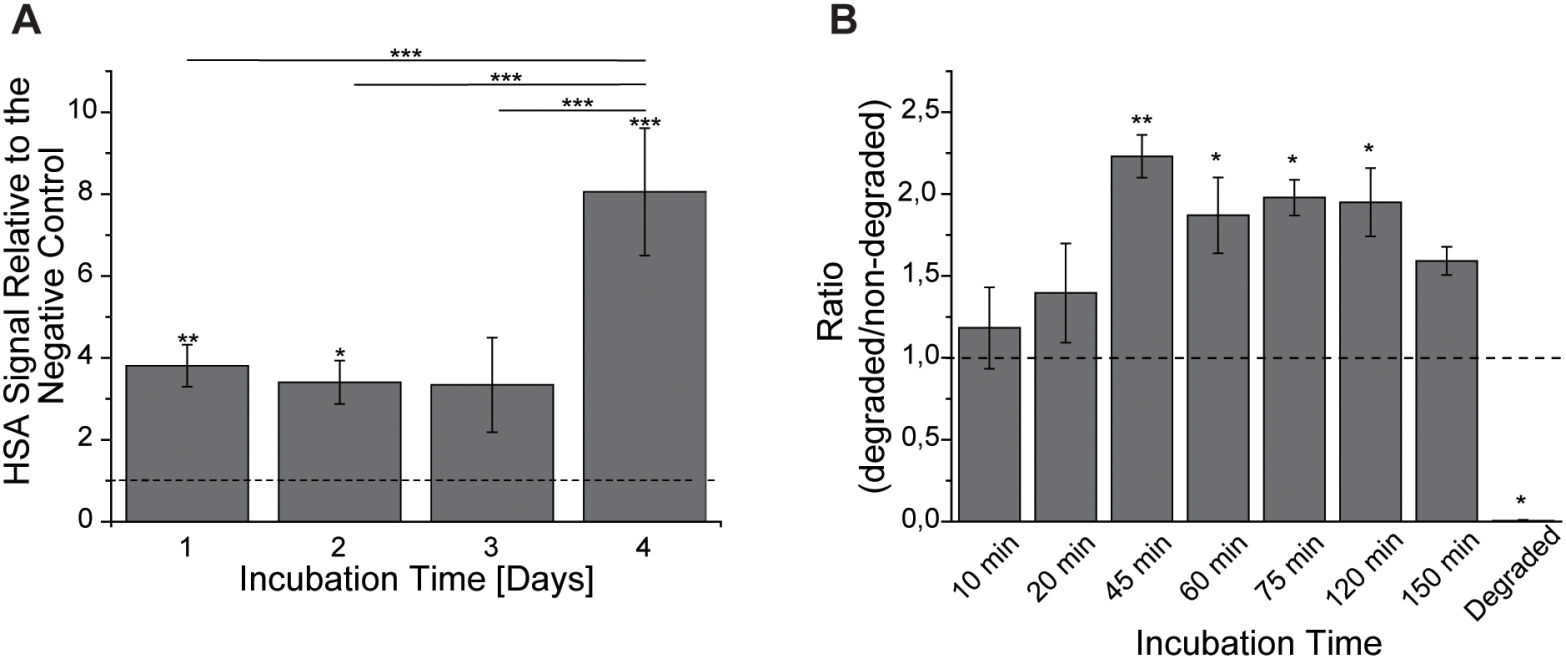
Degradation of pGL3-PEI-coated nanoparticles. **A:** Dot blot immunoanalysis was used to evaluate the internalised HSA amount in Cb cells treated with pGL3-PEI-coated nanoparticles The HSA signal was related to the signal of the negative control (horizontal line). **B:** Similar conditions were used to evaluate the chemical degradation of pGL3-PEI-coated HSA nanoparticles with proteinase K. The HSA signal was related to the signal of non-degraded nanoparticles (horizontal line). Results are shown as mean ± S.E.M. (n = 3).

An important prerequisite for a nanoparticle-mediated delivery system is the biodegradability of the nanoparticles [32]. In order to investigate the degradation kinetics of the pGL3-PEI-coated HSA nanoparticles, the HSA content of total cell lysates from nanoparticle-treated cells was analysed by dot blot. The intensity of the chemiluminescence signal arising from immuno-detection of HSA remained unchanged for the first 3 days of incubation and was followed by an increase on day 4 (Fig. 3A). This increment could result from a higher number of available epitopes for anti-HSA antibodies due to the degradation of the nanoparticles. In order to investigate this, HSA nanoparticles were degraded chemically, and the HSA signal intensities were analysed with dot blot analysis in analogy to the cell samples. With increased degradation time, the HSA signal intensity increased steadily and reached maximum values of ∼2-fold after 45 minutes when compared to non-degraded HSA nanoparticles (*p*<0.01), followed by a decrease below the detection limit after over-night digestion (*p*<0.05) (Fig. 3B).

### *In vitro* transfection of cerebellar cells

**Figure 4:**
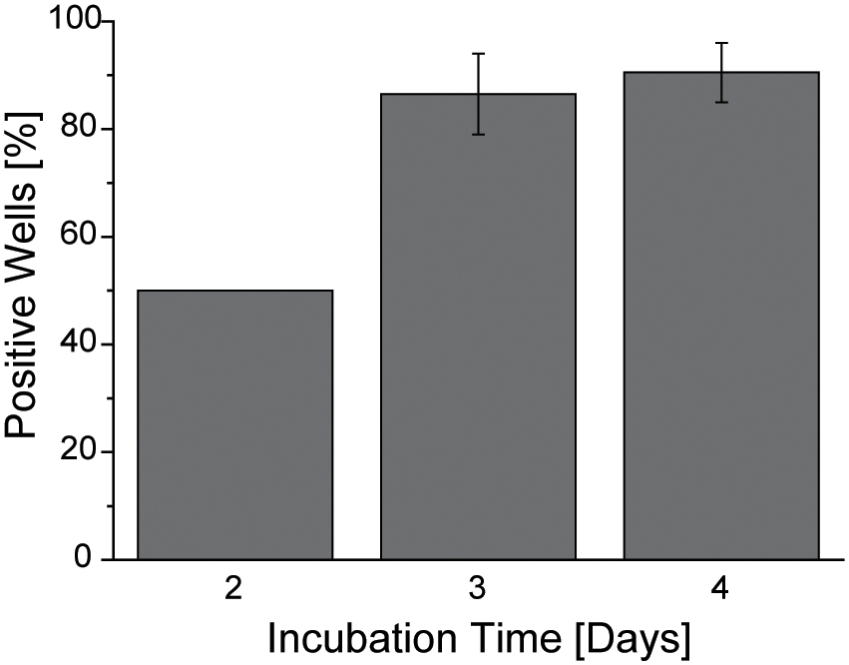
*In-vitro* expression levels of luciferase reporter protein. Cb cells were transfected with pGL3-PEI-coated nanoparticles. The graph shows the percentage of positive wells showing luminescence on day 2, 3, 4 after washing the nanoparticles off. Well values higher than negative control average + 3x standard deviation, were considered positive. Results are shown as mean ± S.E.M. (n = 48).

The feasibility of the nanoparticles-mediated cellular transfection was tested by incubating Cb cells with pGL3-PEI-coated HSA nanoparticles. Cb cells were seeded on 96-well plates and incubated with the aforementioned nanoparticles for 72 h. The plate-reader analysis showed detectable luciferase activity in 50%, 86.5%, and 90.5% of the wells (n = 48) when incubated for other 2, 3, or 4 days, respectively (Fig. 4). To inspect whether the low transfection rate on day 2 correlated with the low metabolism and endocytosis rate of the Cb cells, equal analysis was performed on HeLa cells. Under equal experimental conditions as with the cerebellar cells all analysed wells with HeLa cells showed luciferase activity (n = 48) (data not shown).

## Conclusions

This study provides a newly developed transfection system based on human serum albumin nanoparticles possessing a monodisperse size distribution with negative surface charge, which is important for a subsequent *in vivo* administration of this formulation. The HSA nanoparticles were highly taken up into an *in vitro* Cb cell line via endocytosis and promoted the expression of the luciferase control vector (pGL3) as reporter gene. A linear polyethylenimine-derivative (22 kDa) complexed with pGL3 plasmid was used as nontoxic transfecting coating for nanoparticles to enhance the plasmid’s migration into the nucleus [33]. Between 50% and 90.5% of Cb cell wells treated with the formulation could be transfected while under the same condition 100% of treated HeLa cells wells were transfected, which suggests that the transfection activity may be cell-type dependent. Our *in vivo* and *in vitro* studies revealed the partial degradation of the protein particle system to be time-dependant. Due to possible differentiation and morphological changes of Cb cells resulting from extended culture and incubation times it was not feasible to test for total degradation of the particles *in vivo*.

Dadparvar *et al.* have already shown that HSA nanoparticles are able to enhance drug delivery in an *in vitro* model of the blood-brain barrier [34]. The results of this study encourage the subsequent *in vivo* adaptation of HSA nanoparticles in order to promote gene therapy approaches in the intact brain. However, to improve the brain targeting of nanoparticles and their transport across the blood-brain barrier the addition of targeting molecules, such as apolipoprotein E, A-1, transferrin, or insulin, might be unavoidable [14], [15], [35], [36]. With the use of covalently attached bifunctional crosslinkers, such as the Mal-PEG-NHS, the conjugation presents no significant technological challenge, but provides additional possibilities to adapt the system for wide range of applications.
